# A global picture of pharmacy technician and other pharmacy support workforce cadres

**DOI:** 10.1016/j.sapharm.2016.12.004

**Published:** 2017

**Authors:** Tamara Koehler, Andrew Brown

**Affiliations:** a University of Applied Sciences, Netherlands; b A.N.Brown Health Systems Strengthening Consultancy Pty Ltd, 121/54 Printers Way, Kingston, ACT, Australia

## Abstract

**Introduction:**

Understanding how pharmacy technicians and other pharmacy support workforce cadres assist pharmacists in the healthcare system will facilitate developing health systems with the ability to achieve universal health coverage as it is defined in different country contexts. The aim of this paper is to provide an overview of the present global variety in the technician and other pharmacy support workforce cadres considering; their scope, roles, supervision, education and legal framework.

**Material and methods:**

A structured online survey instrument was administered globally using the Survey Monkey platform, designed to address the following topic areas: roles, responsibilities, supervision, education and legislation. The survey was circulated to International Pharmaceutical Federation (FIP) member organisations and a variety of global list serves where pharmaceutical services are discussed.

**Results:**

193 entries from 67 countries and territories were included in the final analysis revealing a vast global variety with respect to the pharmacy support workforce.

**Roles and competency:**

From no pharmacy technicians or other pharmacy support workforce cadres in Japan, through a variety of cadre interactions with pharmacists, to the autonomous practice of pharmacy support workforce cadres in Malawi.

**Responsibilities:**

From strictly supervised practice with a focus on supply, through autonomous practice for a variety of responsibilities, to independent practice.

**Supervision:**

From complete supervision for all tasks, through geographical varied supervision, to independent practice.

**Education:**

From on the job training, through certificate level vocational courses, to 3–4 year diploma programs.

**Legislation, regulation and liability:**

From well-regulated and registered, through part regulation with weak implementation, to completely non-regulated contexts.

**Conclusion:**

This paper documents wide differences in supervision requirements, education systems and supportive legislation for pharmacy support workforce cadres globally. A more detailed understanding of specific country practice settings is required if the use of pharmacy support workforce cadres is to be optimized.

## 1 Introduction

### 1.1 The sustainable development goals

In 2016 the United Nations transitioned from a focus on the Millennium Development Goals (MDGs) to a renewed focus on international equity through the cross-sectional application of 17 Sustainable Development Goals (SDGs).^1^, ^2^ Although significant progress has been made to address medicines access, inequality continues in many countries and regions of the world.^3^ The lack of sufficient human resources to provide pharmaceutical services is a significant factor in many environments, and often pharmacy technicians and other pharmacy support workforce cadres are used extensively to ensure basic services, as pharmacists are unavailable.^3^, ^4^ This is in contrast to high-income environments where pharmacy technicians and other pharmacy support workforce cadres are working more closely with pharmacists to allow delivery of a greater range of more complex pharmaceutical services.^5^

The SDGs adopted in 2015 aim to ‘end poverty, protect the planet, and ensure prosperity for all’ as part of a new sustainable development agenda focusing on equity.^2^ Each goal has specific targets to be achieved over the next 15 years, with a recognition of the interrelationship between poverty, sustainability and prosperity and a link to health outcomes. Goal 3, increased health and wellbeing, provides the focus for health improvements with the acknowledgment that pharmaceutical service delivery will be essential for progress to be noted in many countries.^2^ Specifically, SDG Goal 3 includes financial risk protection, access to quality essential health-care services and access to safe, effective, quality and affordable essential medicines and vaccines for all.

Understanding how pharmacy technicians and other pharmacy support workforce cadres assist pharmacists in the healthcare system will facilitate developing health systems with the ability to achieve this SDG goal.

### 1.2 Medicines availability, access and people centred health systems

The World health Organization (WHO) published The World Medicines Situation report in 2011 which alarmingly notes that one third of the world's population does not have access to regular life-saving medicines.^3^ This reality sees large numbers of the world population suffering from death and significant morbidities from preventable or treatable diseases such as pneumonia, malaria, HIV/AIDs, tuberculosis, malnutrition and dehydration from diarrhoea.^1^ In many high income countries, aging populations, polypharmacy, use of more complex medicines and rising costs of healthcare is placing pressure on health systems.^6^, ^7^ The pharmacy workforce has a role to play to deal with these pressures.^8^ The International Pharmaceutical Federation (FIP), acknowledges that there is a need to both increase medicines access to patients in low-income environments while also ensure the optimal use of the pharmacist in a variety of clinical settings.^9^, ^10^

### 1.3 Global human resources for health

In 2006, the WHO brought to the world's attention the vast shortage of the human resources required for minimum health service delivery.^11^ At that time an estimated global shortage of 4.3 million health workers was noted, with critical shortages in 57 of the poorest countries in the world. Since that time increased urbanisation, an increased middle class in low- and middle-income countries and an ageing population in many high-income countries has seen increasing demands on health services with a corresponding increase in the number of health workers required to meet this need, including the pharmacy workforce.^12^, ^13^ The FIP 2012 Workforce Report and recent workforce trends report also highlights the shortage of pharmacy workforce cadres in many countries.^8^, ^14^

In 2016, the WHO Health Assembly accepted the ‘Global Human Resources for Health Strategy’.^12^ The strategy includes reference to pharmacy workforce requirements and presents recommendations on how countries may meet their human resources for health (HRH) needs. Within this WHO strategy, mid-level cadres play a significant role in meeting HRH needs due to their wider availability, country specific nature, lower cost and shorter ‘production’ times.^4^, ^15^ WHO describes mid-level providers as ‘health workers with 2–3 years of post-secondary school healthcare training who undertake tasks usually carried out by doctors, nurses, or other health professionals.^16^ Technicians and other pharmacy support workforce cadres are a key subset of mid-level cadres.^12^

### 1.4 FIP guidelines and standards that engage pharmacy support workforce

FIP has had a focus on pharmacy workforce for many years, with the 2009–2012 ‘Pharmacy Education Taskforce’ documenting the global shortage of pharmacists, technicians and pharmacy support workforce cadres in their 2009 and 2012 Global Pharmacy Workforce Reports.^14^, ^17^ Other articles published by experts in this field have further documented the shortage of pharmacists, technicians and other pharmacy support workforce cadres, with an emphasis on exploring local ‘needs-based’ approaches to human resources issues.^18^, ^19^ That is, approaches that consider local issues rather than impose global standards. In 2014 FIP Education Initiatives (FIP*Ed*) introduced a new structure that incorporates pharmacy workforce planning issues and provides the platform for ongoing work in this area by FIP. Under this new structure the ‘Global Pharmacy Workforce Intelligence: Trends Report 2015’ was published.^8^ This report highlighted two workforce issues relevant to technician and pharmacy support workforce considerations:

• The aspiration of many countries towards establishing universal health coverage will require an enhanced health workforce, including pharmacists that can meet the need for pharmaceutical expertise in the population it serves. It is important to monitor trends in the workforce over time.
• There is still much to be done, with some regions and low-income countries still displaying a disproportionately low number of pharmacists or limited overall capacity for delivering pharmacy services.

### 1.5 Roles and scope

**Fig. 1:**
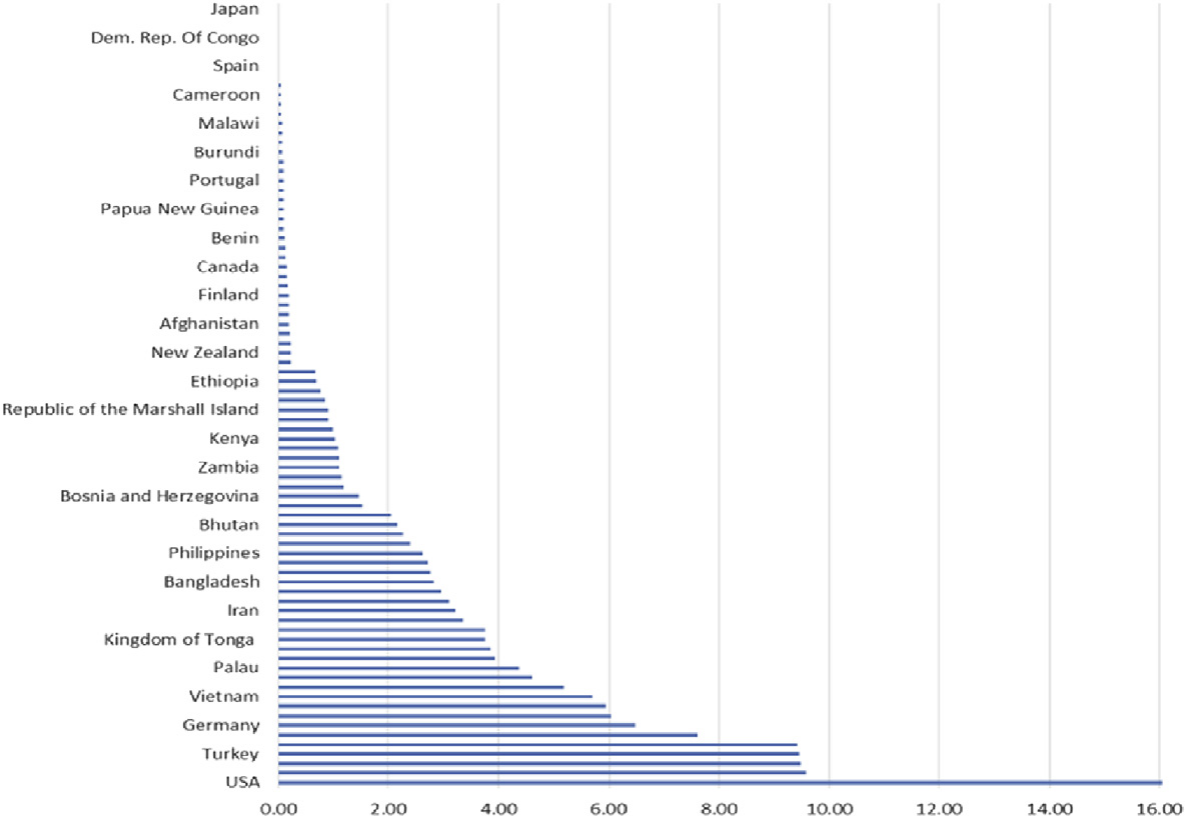
Density of Pharmacy Technicians per 10,000 population (FIP Pharmacy Workforce data 2012, Christopher John, Royal Pharmaceutical Society).

**Fig. 2:**
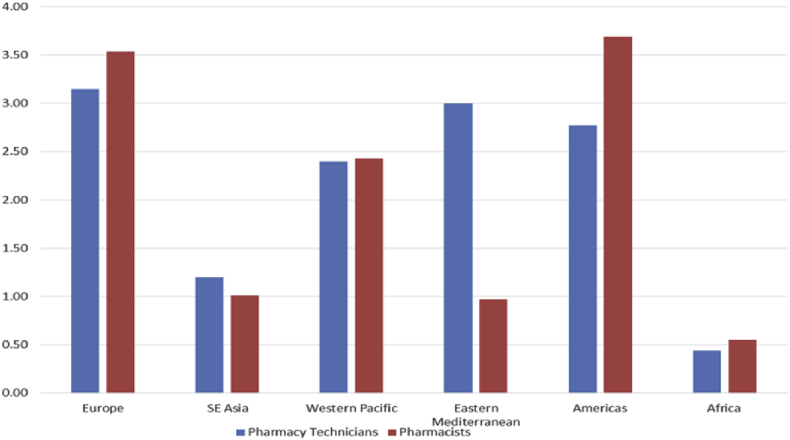
Comparison of pharmacy technician and pharmacist average density per 10,000 population by WHO region (FIP Pharmacy Workforce data 2012, Christopher John, RPS).

FIP 2012 pharmacy workforce data further documents the large variation of numbers of pharmacy support workforce cadres between countries and WHO regions, with low income regions having less pharmacy workforce compared to higher income regions (Fig. 1). It is also interesting to note the large variation in the ratio of pharmacy technicians to pharmacists in different countries (Fig. 2). More detailed data documenting this variation in terms of education, supervision and legislation requirements, is not available.

Globally the roles and scope of work for technicians and pharmacy support workforce cadres vary greatly according to country and practice areas within that country. Clear scope of practice documentation for a variety of pharmacy technician and other pharmacy support workforce cadres exists in many countries but not every country. The general trend is for middle- and high-income countries to have more detailed documentation and legislation supporting all cadres that contribute to the delivery of pharmaceutical services. It is interesting to note that current international labour definitions for pharmaceutical technicians and assistants reflect the core functions of these cadres but do not reflect how these cadres practice in many countries of the world.^20^ For example, registered pharmacy technicians in the United Kingdom and Denmark practice dispensing without the supervision of a pharmacist.^21^

In recent years there have been a number of papers exploring new and expanding roles for the pharmacy support workforce cades. Many of these new roles have been explored with the aim of ‘freeing-up’ the pharmacist, while in countries with few pharmacists these workers have extended roles due to necessity. Table 1 provides a summary of examples of some of these different roles as noted by a variety of publications from different countries.

### 1.6 Objective

The aim of this study was to provide an overview of the present global variety in pharmacy technician and other pharmacy support workforce cadres considering their roles, responsibilities, supervision, education and legal framework.

## 2 Methods

Previous research investigating global aspects of technician and pharmacy support workforce cadres was conducted in 2012 using a structured online survey approved by the University of Canberra ethics committee and administered through the ‘Survey Monkey’ online platform.^37^ The structured survey instrument consisted of closed questions with answer options and open ended questions covering the following topic areas: roles, responsibilities, supervision, education and legislation. The 2012 survey tool was initially trialled with respondents from seven countries (Australia, Laos, New Zealand, Papua New Guinea, Rwanda, Uganda, and United States of America) for reliability and validity.^37^ The survey instrument captures the experiences and perceptions of individuals in their work environments, rather than specific country workforce profiles. In the 2012 survey, further data were collected via oral interviews and more detailed case studies. Triangulation of these three methods produced similar results.^37^

With the aim of this study to gain an overview of the global variety of aspects of pharmacy support workforce cadres, the 2012 tool was considered an appropriate tool (detailed case studies published in this journal issue provide validated country specific data.) The 2012 tool was reviewed by the FIP ‘Pharmacy Support Workforce Technical Working Group’ with minor edits made to contextualise the survey. Specifically, the order of the sections was rearranged to that in Table 2. Extra questions regarding scope and responsibilities were added and education questions expanded to include continuing professional development. The technical working group determined that such changes would not affect the reliability and validity of the survey. A full copy of the revised survey instrument is available here: https://www.surveymonkey.net/r/?sm=0gCNE_2FF_2F4S7rFHQzcg75EM6qyzqalW4RqVQo3kAoEo8_3D.

The survey was circulated to International Pharmaceutical Federation (FIP) member organisations (country based pharmaceutical societies from around the world), through the FIP Office and a variety of global list-servs where pharmaceutical services are discussed [e.g. E-DRUG (global list serve of individuals interested in a range of medicines access issues), International Association of Public Health Logisticians (IAPHL) (a list serve of individuals that have a direct interest in health logistics and supply chain), FIP CoP (a community practice of individuals interested in pharmaceutical education and practice)]. Additional exposure was provided through professional LinkedIn networks. The survey was conducted between October 2015 and January 2016 with a total of three reminder notices provided through the above listservs.

Consistent with the use of online ‘opt-in’ surveys individual consent was assumed when a respondent chose to participate in the survey. Introductory information regarding the purpose of the survey, anonymity and use of survey data was provided.

## 3 Results

### 3.1 Demographics

A total of 193 entries from 67 countries and territories representing each of the WHO regions were included in the final analysis. Of these, 62 (35%) completed the whole survey. These completion results are similar to those experienced by Brown and Bruno in their separate research using similar workforce surveys.^38^, ^39^, ^40^ The data collected represents the individual opinions and experiences of those in their country context. The open nature of the survey did not allow us to verify specific country profiles but the data collectively provides a picture of global variety from the perspective of the pharmacy workforce. Comparison of multiple data points within countries or comparisons across regions was not undertaken.

The full list of countries and number of respondents per country appears in Table 3.

Of those respondents who completed the demographic section (n = 105), the majority of respondents were pharmacists (Table 4) with most respondents having significant experience both in their profession and current role (33% with more than 5yrs experience). Respondents distributed well across the main practice areas of pharmacy, noting that 21 (11%) of participants were from professional associations within the ‘Other’ category, academia (18%), hospital (16%), government ministry or department (14%), and community (12%) (supplementary file 1 participant demographics).

### 3.2 Cadre names

**Fig. 3:**
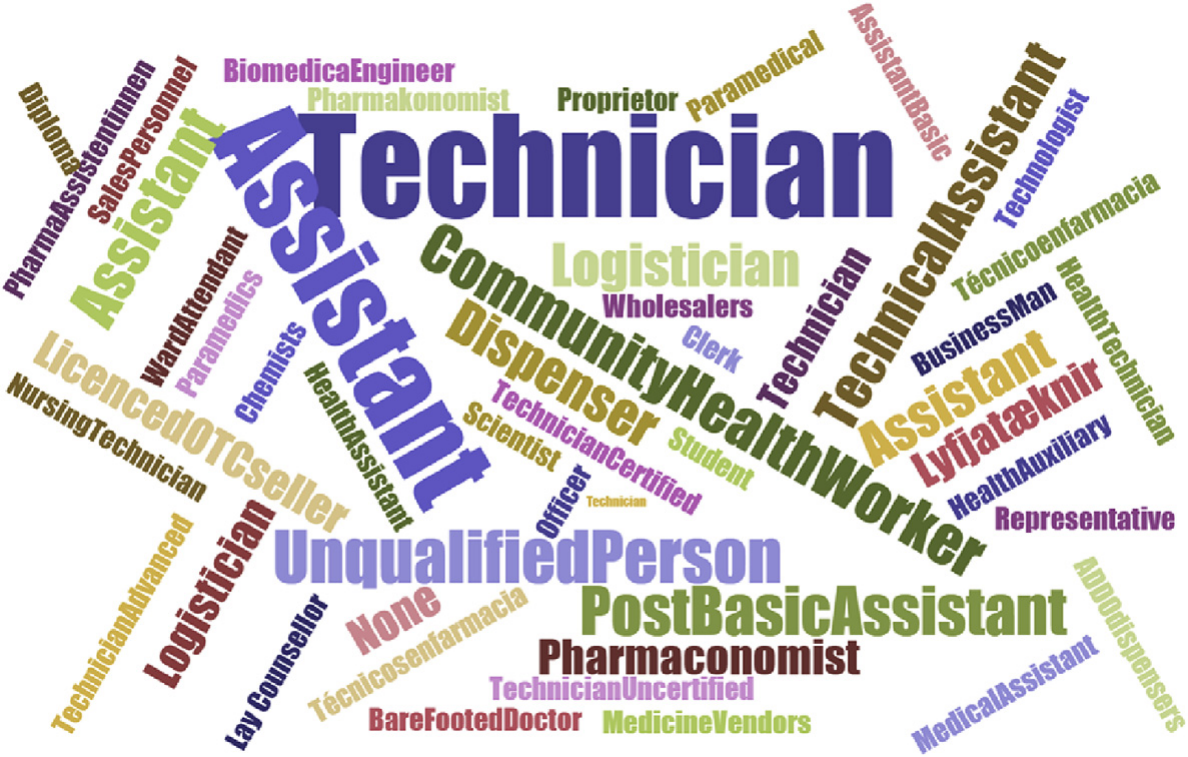
Word cloud documenting the variety of names used globally for pharmacy support workforce cadres emphasising the predominance of the term ‘technician’ and ‘assistant’.^20^

The most frequent name given to the predominant pharmacy support workforce cadre in countries was ‘Technician’, 75%, with a multitude of other titles used across countries (Fig. 3).

Where no technicians or pharmacy support workforce cadres were identified participants noted that this was the case because there were sufficient pharmacists available (Japan), or that there was no existing legislation to support other cadres working in this space, or the current legislation did not reflect what was actually happening in the country context.

### 3.3 Scope

Only 28% (N = 193) of our respondents indicated that a ‘scope of practice document’ existed for the main pharmacy support workforce cadre in that country (10% did not know).

### 3.4 Supervision

There is a global variety in the supervision requirements of technicians and pharmacy support workforce cadres. With 10% (N = 193) of respondents indicating that these cadres work independently all the time, 58% indicating that they work independently some or most of the time and 17% of respondents indicating that technician and pharmacy support workforce cadres never work independently, that is, they are always supervised. Pharmacists (61%, N = 193) were noted as the main supervising cadre.

Respondents (23%, N = 193) indicated that supervision requirements were different in urban compared to rural environments with 30% of respondents indicating unchanged supervision when considering urban rural distribution. A further 15% of participants reported that supervision requirements were ‘somewhat different’ and 8% very different when comparing urban and rural environments.

The most supervised competency area is ‘patient consultation and diagnosis’, while ‘procurement and stock ordering’ was the least supervised competency area (Table 5). Of those competency areas listed in the survey ‘receiving donations of medicines’ and ‘patient consultation and diagnosis’ were competency areas not practiced (not considered relevant to pharmacy support workforce cadres), by one third of respondents (Table 4).

### 3.5 Education

From a global perspective there is significant variety in pharmacy support workforce education approaches with respondents reporting certificate (one to two years) and diploma (three to four years) as the most common, spanning the vocational and academic education pathways. Table 6 further documents the variation around aspects of education.

Public and private institutions both play a significant role in providing education with payment for this education nearly equally distributed between students and other sources (government, employer, donation).

Satisfaction regarding current education approaches was varied with only 32% of respondents noting some degree of satisfaction with the education provided, 10% of respondents were neutral on the issue. When asked ‘how could education for pharmacy support workforce cadres be improved’ the top four responses from 123 respondents were:

• There should be sufficient financial and academic resources available (#16)
• There needs to be a ‘Needs-based’ review of education approaches (#15)
• Education should be more practice based (#11)
• Education approaches need improved accreditation and quality assurance processes (#13)

Quality assurance is the system put in place at a country and or institutional level to ensure that the curriculum content and delivery meets minimum and expected standards.^41^ When asked ‘how could quality assurance (QA) of education for pharmacy support workforce cadres be improved’ respondents noted the following answers (top 4):

• Improve the supervision around implementation and accountability regarding QA processes (#17)
• QA processes for PSW Cadres should be introduced or strengthened (#15)
• Nothing to do as the process is currently working well (#10)
• Improve transparency and feedback during the quality control (QC) process for education (#8)

### 3.6 Legislation, regulation and liability

**Fig. 4:**
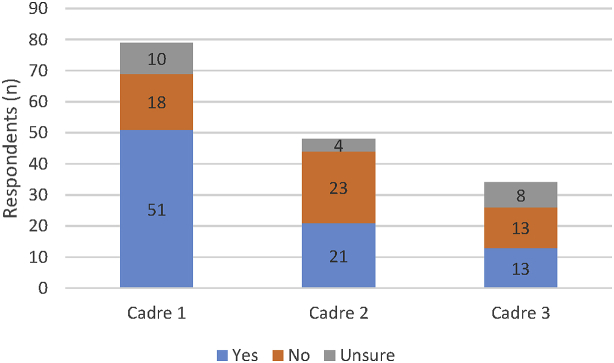
Legislation (i.e. national, state or provincial laws) and regulation (i.e. rules) to frame the practice of each of the national PSW cadres. (N.B. Cadre 1 refers to the ‘main’ PSW cadre as identified by the respondent, with cadre 2 and cadre 3 the next most common PSW cadres.

**Fig. 5:**
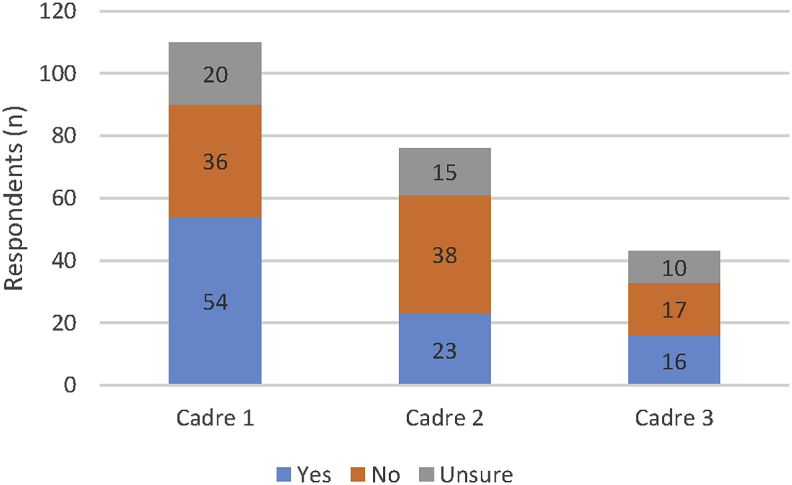
Existence of a defined ‘Scope of Practice’ for each of the national PSW cadres (N.B. Cadre 1 refers to the ‘main’ PSW cadre as identified by the respondent, with cadre 2 and cadre 3 the next most common PSW cadres.

**Fig. 6:**
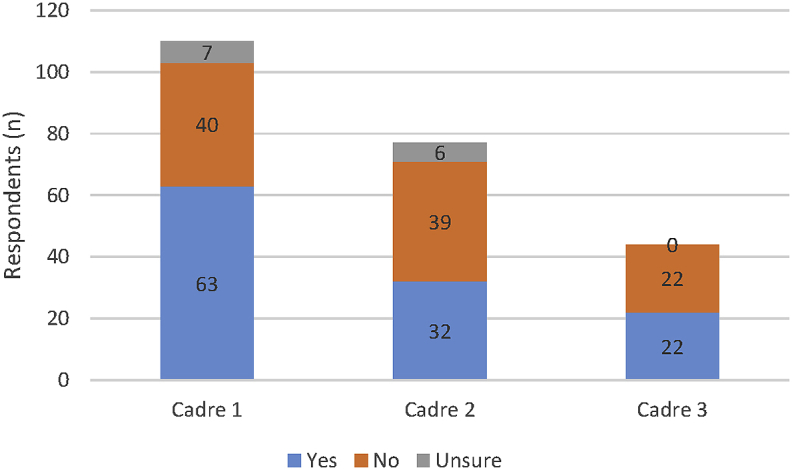
Need of the national PSW cadres to be registered by government in order to work (N.B. Cadre 1 refers to the ‘main’ PSW cadre as identified by the respondent, with cadre 2 and cadre 3 the next most common PSW cadres.

Participants were asked to comment on legislation & regulations (Fig. 4), scope of practice (Fig. 5), and registration (Fig. 6), for up to three PSW cadres present in their country context.

The data again points to significant global variety; from robustly regulated and registered, through part regulation with weak implementation to completely non-regulated contexts. It is interesting to note that cadres apart from those listed as the main pharmacy support workforce cadre are less likely to be regulated by legislation and have reduced requirements for registration. Scope of practice definitions were also reported as being absent by a large number of respondents.

When asked ‘how could legislation and regulatory requirements’ for PSW cadres be improved’ respondents noted the following (top 3):

• Call for registration or re-registration requirements for PSW cadres (#26)
• Review and updating of legislation and regulations (#10)
• Enforcement of legislation (#5)

## 4 Discussion

The results of this survey document the significant global variety of pharmacy technician and pharmacy support workforce cadres globally, in regard to role, scope, supervision, education and regulation. Four different country specific workforce models emerge from the data:

• Countries where there are **no pharmacy support workforce cadres**, only pharmacists, e.g., Japan
• Countries where **pharmacy support workforce cadres are supervised by the pharmacist** by direct or delegated methods, e.g., Australia and South Africa
• Countries where **certain pharmacy support workforce cadres are regulated.** They have accountability to undertake independent practice in a team with pharmacists. (e.g., Canada and Denmark)
• Countries that have weak or outdated legislative structures but where, out of necessity, **pharmacy support workforce cadres work by themselves.** (i.e., current legislation may not reflect actual practice.

### 4.1 Supervision

The supervision of technicians and other pharmacy support cadres may vary in a country depending on geographical location and the availability of other pharmacists or medical cadres. In general, high-income countries (World Bank groupings), with well-developed health systems have strong regulatory systems and sufficient numbers of pharmacists to deliver pharmaceutical services with limited need for non-supervised activity by other pharmacy cadres. In remote locations ‘telepharmacy’ or other remote pharmacist supervised mechanism may be used, where technology, funding and legislation allows (e.g., Kansas (U.S. State)).^42^ There are also a number of countries for example; United Kingdom and Denmark where pharmacy support cadres practice a variety of activities unsupervised in a regulated environment (e.g., final checking of prescriptions after dispensing).

A variety of pharmacy professional associations (e.g., Australia and United Kingdom), have published guidelines to support quality supervision of pharmacy technicians and other pharmacy support workforce cadres.^43^, ^44^

In low-to middle-income countries with less well developed health systems, less funding and a lower number of pharmacists and other medical staff, pharmacy technicians and other pharmacy support workforce cadres take on a greater role in the delivery of pharmaceutical services without supervision. Pharmacists and other medical staff are less likely to be found in rural and remote environments. It is in these environments that other pharmacy cadres often work unsupervised to ensure the delivery of services, even if they are required to do so in urban environments (e.g., South Africa). This may be the case even if there is no legislation or special government policies to support the practice (e.g., Vanuatu and Papua New Guinea).^45^, ^46^ These arrangements are contextually specific within countries and regions of countries depending on the local health needs and availability of staff.

Reports in the literature also discuss a variety of supervision situations. The issue of ‘situational competence’ was documented by Brown in his study of the Vanuatu pharmaceutical system, where across the country he noted that the supervisory circumstances varied according to the availability of staff and the competence they were expected to demonstrate.^38^, ^45^ In the extreme sense, if the driver of the delivery van was the only person in the pharmacy then they would be responsible for dispensing medication directly to patients without further supervision. A similar situation was noted in Papua New Guinea.^46^ The overriding principle was that pharmaceutical service delivery to patients and clinics must go on irrespective of the cadres that were present in the pharmacy section. Results from this current study suggest that this is a more global phenomena, especially in low- and middle-income country contexts. Indeed, ‘situational competence’ can be expected in any country experiencing a shortage of pharmaceutical workforce cadres.^13^, ^14^

### 4.2 Education

FIP encourages a ‘needs-based’ approach to pharmaceutical education where the services required to be delivered in a context are locally determined, the competencies required for specific cadres who deliver these services are agreed and education approaches are developed to meet local development needs for these competencies.^19^, ^47^ Further, FIP suggests guarding against seeking globally standardised curriculum approaches, noting that any country education efforts for the pharmaceutical workforce must be relevant to local service delivery.^19^, ^48^ Efforts taken to apply this approach to the pharmacy support workforce cadres of small island states of the Pacific Islands is one example where excellent engagement and local acceptability can be demonstrated.^49^

For any extended role for pharmacy technicians and pharmacy support workforce cadres to be effective the education requirements must change to meet this new need. The USA Report of the ‘2013–2014 Professional Affairs Standing Committee: advancing the pharmacy profession together through pharmacy technician and pharmacy education partnerships’ notes this in detail and suggests that education institutions should consider forming effective collaborations to ensure appropriate education, training, and certification of pharmacy technicians.^50^ This USA report was tabled, noting a call for improved accountability regarding pharmacy technician education in 2011.^51^

Other countries are also pursuing this aim, including South Africa who has recently reviewed the roles of pharmacy support workforce cadres and related education requirements.^52^, ^53^, ^54^ An overview of the approach taken to educate pharmacy technicians cadres in the United States was recently published, where a state based system is in force.^55^ The paper notes that rigorous debate and discussion is needed regarding the future of pharmacy technician roles and the training required fitting those roles. The United Kingdom takes a wider view when it comes to the education of pharmacy technicians and has published a robust review of education and training with a view to ensuring it was aligned with the changing scope of practice in that context.^56^, ^57^ Intense discussion regarding the appropriateness of pharmacy support workforce training in Germany formed part of the ‘Future congress’ (a national pharmacy congress held in Germany in 2015), and reflects a robust review of roles and associated supportive education structures.^58^

### 4.3 Legislation

In 2010, the Global Health Workforce Alliance (GHWA) released a report summarising the importance of mid-level cadres in meeting healthcare needs. This paper suggests that all who practice health related competencies, including pharmacy technicians and pharmacy support workforce cadres, should work under and be held accountable by appropriate legislation.^16^ Registration of healthcare workers further aids in ensuring accountability and patient protection in the healthcare sector, but as our data shows, registration of pharmacy technicians and other pharmacy support workforce cadres is not routine across countries, with even OECD countries (Organization for Economic Cooperation and Development) such as Australia and Spain, not requiring the registration of these cadres.

### 4.4 Future research

The data presented in this paper presents an introductory overview regarding the variety of roles, responsibilities, supervision, education and legislation that governs pharmacy technicians and other pharmacy support workforce cadres around the world. Research into specific country jurisdictions and comparisons between regions may provide insights into optimal use of pharmacy support workforce cadres and improve patient care.

### 4.5 Limitations

Using a web-based survey limited the data collected to those who had access to a computer and were members of one of the organisations or listservs through which the survey was circulated. Denominator values are not publicly available for the associations and listservs used and so percentage response statistics cannot be calculated. A total of 193 responses would be considered a small number compared to the anticipated global numbers within the pharmaceutical workforce numbers but the data presented provides some insight into the variety of pharmacy support workforce cadres globally, which was the aim of this survey.

## 5 Conclusion

The data presented through this research documents a variety of roles and responsibilities for pharmacy technicians and other pharmacy support workforce cadres globally, with wide differences in supervision requirements, education systems and supportive legislation noted. A more detailed understanding of specific country practice settings is required if the use of pharmacy support workforce cadres is to be optimized.

## Figures and Tables

**Table 1:** Studies on different roles for the pharmacy support workforce.

Year	Practice area	Role or service	Country	Ref.
2009	Hospital	Facilitation of admission and discharge of elderly patients	Portugal	^22^
2012	Hospital	Hospital ward stock top up service	Denmark	^23^
2012	Hospital	Check Tech Check	United States	21, 24
2013	Hospital	Clinical pharmacy technician roles	United States	^25^
2013	Community	Medication therapy management	United States	^26^
2013	Hospital Community	Medication Reconciliation	United States	27, 28
2013	Hospital	Contributing to geriatric treatment in bed-wards	Denmark	^29^
2014	Community	Vaccine advocacy	United States	^30^
2014	Medical center	Medication history taking	United States	^31^
2014	Community	In home medication therapy	United States	^32^
2014	Hospital	Subacute aged care ward service	Australia	^33^
2014	Health center	Enhanced cadre of pharmacy assistants for health centers	Malawi	34, 35
2014	Community	Community pharmacy-based adherence programs	Multiple	^36^

**Table 2:** Summary of survey areas, purpose and example questions.

Survey topic area	Purpose	Example questions
1. Instructions and overview	Orientate the participant to the purpose and scope of the survey	N/A
2. Roles and names	Capture the range of titles that are attributed to pharmacy support workforce cadres internationally	In the country that YOU work, NOT including pharmacists, nurses and doctors, who are the MAIN CADRES providing pharmaceutical services in your country?
3. Supervision	Explore the extent of supervision, by whom and variations with respect to competency type and geography	Does the Pharmacy Support Workforce (PSW) cadres you have identified work independently, without face to face supervision, on a day to day basis? What are the main competences that require supervision for the PSW cadre you have identified? Briefly describe the circumstances (e.g. regulations, geographical, health facility type etc.) where different PSW cadres are allowed to work independently in your country.
4. Education	Explore the range, satisfaction and quality assurance aspects of education	What is the minimum expected level of education that is required for PSW cadres in your country? Overall, are you satisfied with the PSW education which occurs in your country? Please briefly outline the quality assurance procedures for the PSW education in your country.
5. Regulation, registration and scope of practice	Capture the scope and legal requirements for PSW cadres	For each of the cadres you have identified, does a defined 'SCOPE of Practice' exist? For each of the cadres you have identified do they need to be REGISTERED by the government in order to work?
6. Demographics	Understand the profile of the participants who took the survey	Standardised questions were used

**Table 3:** List of countries and number of respondents per country.

Country	Number of respondents	Country	Number of respondents	Country	Number of respondents
Afghanistan	1	Iceland	2	Slovenia	1
Algeria	2	India	7	Solomon Islands	1
Australia	4	Iraq	2	South Africa	17
Belgium	1	Ireland	3	Spain	6
Botswana	1	Japan	2	Sri Lanka	2
Brazil	4	Kenya	1	Sudan	2
Cameroon	1	Lebanon	2	Swaziland	1
Canada	3	Malawi	4	Sweden	1
China	1	Malaysia	3	Switzerland	3
China Taiwan	2	Mongolia	1	Thailand	2
Croatia	3	Morocco	2	Tonga	1
Democratic Republic of the Congo	6	Nepal	4	Uganda	3
Denmark	3	Netherlands	2	Ukraine	1
Dominican Republic	1	New Zealand	5	United Arab Emirates	3
Ecuador	1	Nigeria	10	United Kingdom	9
Ethiopia	3	Norway	1	United Republic of Tanzania	5
Fiji	3	Pakistan	6	United States of America	14
France	1	Papua New Guinea	1	Vanuatu	1
Germany	3	Philippines	1	Viet Nam	1
Ghana	5	Portugal	1	Zambia	2
Greece	1	Romania	1	Zimbabwe	2
Grenada	1	Senegal	1		
Hungary	1	Sierra Leone	1		

**Table 4:** Respondents' profile.

Senior medical professionals	Pharmacist	Pharmacy assistant/technician	Managerial/leadership role	Administrative service professionals	Other	No response
3 (2%)	72 (37%)	7 (4%)	13 (7%)	4 (2%)	6 (3%)	88 (45%)

**Table 5:** The main competency areas that require supervision for the main PSW cadre in respondent's country.

What are the competences that require supervision for the main PSW cadre in your country? (N = 193)									
Answer Options	Full supervision		Some supervision		No supervision		Competency area NOT practiced		Not answered
Procurement (Stock Ordering)	40	21%	69	36%	41	21%	11	6%	32
Receiving donations of medicines	33	17%	42	22%	24	12%	62	32%	32
Distribution of medicines to facilities	47	24%	68	35%	34	18%	11	6%	33
Packing/repacking of medicines	44	23%	70	36%	33	17%	13	7%	33
Disposal of medicines	49	25%	65	34%	32	17%	15	8%	32
Budget and Reimbursement	46	24%	44	23%	22	11%	43	22%	38
Giving medicines information and advice to patients	55	28%	57	30%	30	16%	19	10%	32
Health promotion of non-medicine strategies	34	18%	67	35%	32	17%	29	15%	31
Patient consultation and diagnosis	66	34%	27	14%	16	8%	50	26%	34
Taking a medication history of patients, including ‘medication reconciliation’	53	27%	45	23%	19	10%	44	23%	32
Dispensing medicines to patients	57	30%	62	32%	29	15%	13	7%	32
Reconstituting of medicines	60	31%	50	26%	26	13%	23	12%	34
Preparation or compounding of medications	64	33%	52	27%	25	13%	20	10%	32
Consult with other healthcare professionals	44	23%	60	31%	26	13%	31	16%	32
Checking prescriptions	61	32%	49	25%	27	14%	23	12%	33

**Table 6:** Variation around aspects of education.

What is the minimum expected level of education that is required for the main PSW cadre in your country?						
Type of education?						
No education required	Work based education	Certificate level (vocational)	Diploma level	Degree level (academic)	Other	Not Answered
7 (4%)	9 (5%)	42 (22%)	40 (21%)	14 (7%)	8 (4%)	73

Length of education? (choosing the most relevant time frame)					
<1yr	<2ys	<3yrs	<4yrs	>4yrs	Not answered
20 (10%)	22 (11%)	43 (22%)	16 (8%)	9 (5%)	83

What type of institution mainly gives this education?							
Non Govt. Org.	Govt.	Public technical college (vocational)	Private technical college (vocational)	Public university	Private university	Other	Not answered
8 (4%)	13 (7%)	36 (19%)	18 (9%)	13 (7%)	4 (2%)	19 (10%)	82

Who has the main responsibility for paying for the education?					
Student	Govt.	Non govt. org.	Employer	Other	Not answered
45 (23%)	27 (14%)	2 (1%)	20 (10%)	6 (3%)	93
