# Supplementary material for: A global picture of pharmacy technician and other pharmacy support workforce cadres
Source: Res Social Adm Pharm. 2017 Mar-Apr;13(2):271–9. doi: 10.1016/j.sapharm.2016.12.004 (PMC5317197; doi:10.1016/j.sapharm.2016.12.004)
Supplement: Supplementary file 2 [file mmc2.pdf]

Copy of FIP 2015 Global survey on Pharmacy Technician & Pharmacy Support Workforce

1. Instructions and Overview.

Dear reader,

Thank you for taking part in this research on Pharmacy Support Workforce.
This survey should take approximately 20 to 30 minutes to complete.

Your answers will help build a global picture of the Pharmacy Technician workforce and the Pharmacy Support Workforce, both involved in pharmaceutical services delivery besides pharmacists. This survey will provide information for us to better understand the situation and needs of the Pharmacy Support Workforce internationally.

As you move through this survey you will be asked a series of questions about:
- The names of cadres (= group of people educated for a particular purpose) of staff who provide pharmaceutical services,
[Think about all staff involved in the procurement (purchase), distribution, supply and dispensing of medications in your country]
- Their supervision
- Their education
- Their regulation

The survey design has been approved by the Human Ethics Committee of the University of Canberra.

* 1. In which country do you currently work?

	Country
Please select one	

Other (please specify)

Please answer all following questions in relation to this answer.

2. Cadres (=group of people) providing delivery of pharmaceutical services, inc. supply of medicines

For the following question:

1. Please take your time and list all the cadres, or job descriptions you are aware of that provide pharmaceutical services but are NOT pharmacists.
2. Think about both the private & public sectors and community & hospital practice.
3. Give the exact title for the role where possible.
4. Think about all staff involved in the procurement, distribution, supply and dispensing of medications.
5. Place each title on a new line.
6. The titles you choose here will reappear through out the rest of the survey.

* 1.) In the country that YOU work, NOT including pharmacists, nurses and doctors, who are the MAIN CADRES providing pharmaceutical services in your country?

2.)

3.)

4. If there are more than three cadres, please list the additional titles here.

5. If there is NO Pharmacy Support Workforce in your country, please comment on what you think are the reasons for this?

6. Of the three cadres you previously described (which appear again below), which cadre forms the largest group within the Pharmacy Support Workforce?

- "[Q2]"
- "[Q3]"
- "[Q4]"

3. Supervision of the different Pharmacy Support Workforce cadres

The questions on "Supervision" will be repeated for the different cadres you described earlier in the questionnaire.

* 1. Does the "[Q2]" work independently, without face to face supervision, on a day to day basis?

- ◯ Always
- ◯ Most of the time
- ◯ Some of the time
- ◯ Never

2. What are the main competences that require supervision for the "[Q2]" you have identified ?

	Full supervision	Some supervision	No supervision	Item not applicable in my country
Procurement (Stock Ordering)	◯	◯	◯	◯
Receiving donations of medicines	◯	◯	◯	◯
Distribution of medicines to facilities	◯	◯	◯	◯
Packing/repacking of medicines	◯	◯	◯	◯
Disposal of medicines	◯	◯	◯	◯
Budget and Reimbursement	◯	◯	◯	◯
Giving medicines information and advice to patients	◯	◯	◯	◯
Health promotion of non medicine strategies	◯	◯	◯	◯
Patient consultation and diagnosis	◯	◯	◯	◯
Taking a medication history of patients, including 'medication reconciliation'	◯	◯	◯	◯
Dispensing medicines to patients	◯	◯	◯	◯
Reconstituting of medicines	◯	◯	◯	◯
Preparation or compounding of medications	◯	◯	◯	◯
Consult with other healthcare professionals	◯	◯	◯	◯
Checking prescriptions	◯	◯	◯	◯

* 3. What is the title of the person responsible for supervising the "[Q2]"?

[ ]

4. Briefly describe the circumstances (eg regulations, geographical, health facility type etc) where different cadres within pharmacy support workforce are allowed to work independently in your country.

[ ]

4. Differences in "Situational Competences" and "Supervision" due to work area and facility.

By "Situational Competencies" we mean that different competencies may be asked of an individual depending on the location of their work or depending on which other staff are available at a particular time.

* 1. Is there a difference in the overall EXPECTED COMPETENCES of cadres within the Pharmacy Support Workforce in URBAN or RURAL areas in your country?

- ◯ Exactly the same
- ◯ Mostly the same
- ◯ Somewhat different
- ◯ Very different

Please briefly comment on your response

* 2. Is there a difference in the overall SUPERVISION LEVELS of cadres within the Pharmacy Support Workforce in URBAN or RURAL areas in your country?

- ◯ Exactly the same
- ◯ Mostly the same
- ◯ Somewhat different
- ◯ Very different

Please briefly comment on your response

* 3. Is there a difference in the overall EXPECTED COMPETENCES of cadres within the Pharmacy Support Workforce between HOSPITAL, PRIMARY HEALTHCARE FACILITIES or COMMUNITY PHARMACIES in your country?

- ◯ Exactly the same
- ◯ Mostly the same
- ◯ Somewhat different
- ◯ Very different

Please briefly comment on your response

* 4. Is there a difference in the overall SUPERVISION LEVELS of cadres within the Pharmacy Support Workforce in HOSPITAL, PRIMARY HEALTHCARE FACILITIES or COMMUNITY PHARMACIES in your country?

- ◯ Exactly the same
- ◯ Mostly the same
- ◯ Somewhat different
- ◯ Very different

Please briefly comment on your response

Copy of FIP 2015 Global survey on Pharmacy Technician & Pharmacy Support Workforce

5. Education

The questions on "Education" will be repeated for the different cadres you described earlier in the questionnaire.

1.

What is the minimum expected level of education that is required for the "[Q2]" cadre in your country?

	Type of education?	Length of education?	What type of institution mainly gives this education?	Who has the main responsibility for paying for the education?	edu[illegible]
Please choose the best answer					

If you choose other (please specify)

* 2. Please specify the title of the education occurs for the "[Q2]" cadre in your country.

* 3. Please mention any prerequisites, or necessary requirements, a student must have before starting that education?

* 4. Overall, are you satisfied with the Pharmacy Support Workforce education which occurs in your country?

- ◯ Satisfied
- ◯ Slightly satisfied
- ◯ Neither satisfied nor dissatisfied
- ◯ Slightly dissatisfied
- ◯ Dissatisfied

* 5. What do you like most about the education that occurs?

* 6. What do you like least about the education that occurs?

* 7. What improvements could be made to the education?

Copy of FIP 2015 Global survey on Pharmacy Technician & Pharmacy Support Workforce

6. Quality Assurance of Education

Quality Assurance is the maintenance of a standard in Pharmacy Support Workforce education. In some countries this may be done by a specific health professional board for example a Pharmacy Board, in others it may be the responsibility of a Government department such as the Ministry of Health or the Ministry of Education. Some countries don't have any such quality assurance process for education or training.

* 1. Please briefly outline the Quality Assurance procedures for the Pharmacy Support Workforce education in your country.

* 2. Are the Quality Assurance procedures for the pharmacy support workforce education;

	Yes	No	Unsure
A Government Initiative	◯	◯	◯
Endorsed by the whole profession	◯	◯	◯
Developed by all the stakeholders involved (including students)	◯	◯	◯
Evidence based	◯	◯	◯
Validated by reliable outcomes/measures	◯	◯	◯
Publicly disclosed	◯	◯	◯
Reviewed and updated regularly	◯	◯	◯

* 3. Overall, are you satisfied with the Quality Assurance of education which occurs?

- ◯ Satisfied
- ◯ Slightly satisfied
- ◯ Neither satisfied nor dissatisfied
- ◯ Slightly dissatisfied
- ◯ Dissatisfied

* 4. What improvements could be made to the Quality Assurance of the pharmacy support workforce education?

7. Regulation, Registration and Scope of practice

* 1. For each of the cadres you identified within the Pharmacy Support Workforce is there LEGISLATION (national, state or provincial laws) and REGULATIONS (rules) to support their practice?

	Yes	No	Unsure
[Q2]	○	○	○
[Q3]	○	○	○
[Q4]	○	○	○

Please provide 'web links' to any relevant LEGISLATION or REGULATIONS here

* 2. For each of the cadres you have identified, does a defined 'SCOPE of Practice' exist?

	Yes	No	Unsure
[Q2]	○	○	○
[Q3]	○	○	○
[Q4]	○	○	○

Please provide a 'web link' to any available 'SCOPE of Practice' documents here

* 3. For each of the cadres you have identified do they need to be REGISTERED by the government in order to work?

	Yes	No	Unsure
[Q2]	○	○	○
[Q3]	○	○	○
[Q4]	○	○	○

* 4. For each of the cadres you have identified do they need to be RE-registered every few year in order to work?

	Yes	No	Unsure
[Q2]	○	○	○
[Q3]	○	○	○
[Q4]	○	○	○

* 5. What are the requirements for re-registration?

* 6. What improvements could be made to these regulations and their enforcement?

Copy of FIP 2015 Global survey on Pharmacy Technician & Pharmacy Support Workforce

8. Demographics

* 1. What is your profession?

Please select one.

Other (please specify)

* 2. Where is your main place of work?

3. How long have you worked;

	Time
At your current Facility?	
In your current Professional Role?	

4. What is the highest level of school you have completed or the highest degree you have received?

- ◯ Less than high school degree
- ◯ High school degree or equivalent (e.g., GED)
- ◯ Some college but no degree
- ◯ Associate degree
- ◯ Bachelor degree
- ◯ Post Graduate degree (Certificate, Master, PhD)

* 5. In a few words, please state your main day to day duties/responsibilities at work?

6. This question is OPTIONAL

Many organisations, including the World Health Organisation, are unable to use data, such as the information this survey will generate, unless the contact details of the sources are provided.

Any data that is used will be de-identified.

Please consider providing your contact details in the space below, They will ONLY be used for the purpose of validation by the research team if required, and will NOT be shared with any other parties.

9. Thank you

The Pharmacy Support Workforce Technical Working Group of the International Pharmaceutical Federation would like to thank you for taking the time to fill in this survey.

If you have any inquires regarding this research please direct them to:

Dr XXXXXXXXXX

1. If you would consider taking part in a telephone interview or online discussion on the findings of this survey, please leave your details below.

You will not be contacted by us except for the stated purpose.
Your name, phone number or email address will not be passed on to any other source.

Name

Email Address

Phone Number (please include country code)

Is there a convenient time to contact you?

2. Please use this space if you have any further comments
